# ASCA-YOLO: Adaptive Sparse and Context-Aware YOLO Algorithm for Forest Wildfire Detection

**DOI:** 10.3390/s26113444

**Published:** 2026-05-29

**Authors:** Yu Hao, Kangning Wang, Li Zhang, Zibo Yuan

**Affiliations:** 1 School of Airspace Science and Engineering, Shandong University, Weihai 264209, China; 202300800204@mail.sdu.edu.cn (Y.H.); 202300800266@mail.sdu.edu.cn (K.W.); 2 Shandong Key Laboratory of Intelligent Electronic Packaging Test and Application, Shandong University, Weihai 264209, China; 3 Discipline Inspection and Supervision School, Shandong University, Weihai 264209, China; 202300630090@mail.sdu.edu.cn

**Keywords:** wildfire detection, UAV, YOLO26, multi-scale feature representation, contextual saliency attention, IoU-based loss

## Abstract

**Highlights:**

**What are the main findings?**

- ASCA-YOLO is developed, integrating FWAMSConv, FWSCSAttention, and FWASIoU for UAV-based wildfire detection.
- The model significantly improves the detection of small, sparse fire targets and reduces background false alarms.

**What are the implications of the main findings?**

- The method achieves highly accurate and stable localization for non-rigid targets like dynamic fire and smoke.
- It offers an excellent balance between detection quality and computational cost, proving highly suitable for real-time edge deployment on UAVs.

**Abstract:**

Combining Unmanned Aerial Vehicle (UAV) remote sensing with computer vision has become an efficient approach to detect forest wildfires. Nevertheless, existing methods still face several challenges, including missed detection of small fire spots and slender smoke under limited computational resources, false alarms caused by complex forest backgrounds, and insufficient adaptability to the irregular and dynamic morphology of fire and smoke. To address these issues, an improved YOLO26-based model, termed ASCA-YOLO, is proposed. Specifically, FWAMSConv module is introduced to improve multi-scale indicator representation of small and sparse targets. In addition, FWSCSAttention mechanism is designed to reduce background interference by modeling contextual feature distributions. Moreover, FWASIoU loss is developed to improve bounding box regression for non-rigid targets. The experimental evaluation indicates that, relative to YOLO26, the proposed model decreases the parameter count and FLOPs by 19.2% and 21.3%, respectively. Meanwhile, recall reaches 0.809 and precision reaches 0.870, indicating improved detection performance under complex conditions. In addition, mAP50-95 is improved by 12.9%, reflecting more stable localization for irregular wildfire targets. Overall, ASCA-YOLO attains a better equilibrium between detection quality and computational cost than several mainstream object detection models, indicating its potential for real-time UAV-based wildfire monitoring.

## 1. Introduction

Forests are critical ecosystems that provide essential services, including biomass production, water conservation, carbon sequestration, and biodiversity preservation [1,2]. However, they are increasingly affected by wildfires [3]. According to recent statistics, carbon emissions from forest wildfires reached about 2.2 Pg throughout the 2024–2025 worldwide wildfire period [4]. Additionally, large-scale wildfire events have released substantial amounts of toxic particulate matter and chemical pollutants, causing long-term environmental impacts on the atmosphere, water, and soil systems [5]. Wildfires also impose considerable direct and indirect economic losses [6,7]. Therefore, developing accurate and efficient early wildfire detection methods is important for mitigating fire spread, protecting ecological systems, and reducing socio-economic impacts.

Conventional forest fire monitoring approaches are mainly based on ground sensors and Internet of Things (IoT) techniques for early warning. Through the deployment of wireless sensor networks [8] and air quality sensors [9], these systems can support real-time environmental data acquisition and fire warning [10]. Some studies have also incorporated infrared sensing to build multimodal ground monitoring systems [11]. For large-area observation, satellite remote sensing is frequently adopted because of its wide coverage. Multi-source satellite images can be used to continuously track wildfire dynamics and assist large-scale fire assessment [12,13]. However, in complex terrain, ground-based systems often require high deployment and maintenance costs and may still leave monitoring blind areas. Meanwhile, satellite-based approaches are constrained by revisit intervals and spatial resolution. Consequently, both approaches still have difficulty achieving real-time detection and identifying small fires at an early stage.

To address these shortcomings, an increasing number of recent studies have investigated wildfire detection by integrating UAV remote sensing with computer vision techniques [14]. These approaches offer flexible deployment and high-resolution observations and therefore present clear advantages over traditional large-scale monitoring methods [15]. They also enable automated early detection at relatively low cost. Earlier studies in this field mainly depended on conventional machine learning methods, in which classifiers were usually built from handcrafted indicators including flame spectral, smoke texture, or environmental thresholds. For example, earlier studies explored several vision-based strategies for fire detection. Ko et al. [16] combined visual sensors with an SVM classifier, while Chitade et al. [17] focused on fire segmentation using color-based k-means clustering. Habiboğlu et al. [18] investigated video fire detection with covariance matrices and reported faster performance than earlier methods. A broader comparison of rule-based and machine learning-based approaches was later provided by Toulouse et al. [19]. Dampage et al. [20] integrated environmental sensor data into a regression-based detection framework, whereas Yang et al. [21] proposed a Preferred Vector Machine (PVM) model to enhance detection accuracy and reduce false alarms. However, these methods depend strongly on handcrafted features, which are often less robust in complex forest environments and insufficient for learning deep spatial representations. As a result, their ability to capture minor fires in the initial phase remains limited, which can lead to missed detections and false alarms [19,21].

Recent progress in deep learning has made it possible to automatically extract features through convolutional neural networks (CNNs), thereby reducing dependence on manual feature engineering. Two-stage object detection methods, including Fast R-CNN [22], Mask R-CNN [23], and Faster R-CNN [24], depend on region proposal mechanisms and therefore usually involve high computational cost and inference latency. Such characteristics make these models difficult to deploy on UAV platforms with limited computing resources. By comparison, one-stage object detection methods like YOLO [25] and SSD [26] are generally faster at inference because they predict object locations and categories in a single pipeline. Their speed advantage is particularly useful for UAV-based wildfire monitoring, where real-time response is important. Recently, approaches driven by deep learning have been extensively deployed to wildfire detection. Jiao et al. [27] designed a YOLOv3-tiny algorithm for detecting fire on limited datasets. Hung et al. [28] optimized detection performance by applying data augmentation and using a Deep Normalized Convolutional Neural Network (DNCNN). Models based on the Transformer architecture have also been introduced to capture global contextual information. For example, Ghali et al. [29] introduced a Transformer-based detection and segmentation architecture, while Qiao et al. [30] introduced FireFormer to reduce false alarms. Liu et al. [31] advanced TFNet to improve multi-scale feature fusion. However, Transformer-based models often involve high computational complexity. To address this issue, recent studies have focused on lightweight model design. Liu et al. [32] proposed MCAN-YOLO based on YOLOv7, while Han et al. [33] introduced LUFFD-YOLO based on YOLOv8n. Jin et al. [34] developed SWVR by incorporating GSConv, and Zhu et al. [35] proposed YOLO-MP to improve efficiency for edge deployment. Recent work has also focused on improving model performance in challenging environments. For example, Zhou et al. [36] studied robustness to occlusion, while Guo et al. [37] dealt with scale variation through state space modeling. Han et al. [38], in turn, introduced a multitask framework to better handle extreme conditions.

Despite these developments, current models still show two major limitations in practical UAV-based wildfire detection. First, accurately perceiving small and sparse targets remains difficult when computational resources are limited. From high-altitude UAV viewpoints, fire spots and smoke usually appear at very small scales. After model compression, deep features may be weakened during downsampling, which can result in the absence of critical target features. Consequently, accurate detection of early-stage fires becomes difficult. Second, existing models remain limited in robustness within complex forest environments and are not sufficiently adaptable to non-rigid targets. Background elements with fire-like visual characteristics can introduce false detections during feature extraction. Moreover, conventional attention mechanisms and bounding box regression strategies do not handle the dynamic and irregular morphology of wildfire spread well. This may result in unstable feature representations and lower localization accuracy.

To tackle these limitations, this study develops an improved YOLO26-based wildfire detection model, termed ASCA-YOLO. The major contributions of this paper are summarized as follows:

(1) A Forest Wildfire Adaptive Multi-Scale Convolution (FWAMSConv) module is proposed to strengthen the extraction of multi-scale features for small and sparse targets while preserving computational efficiency.
(2) A Forest Wildfire Sparse Contextual Saliency Attention (FWSCSAttention) mechanism is designed to characterize contextual feature distributions and suppress background interference.
(3) A Forest Wildfire Adaptive Sparse-Aware IoU (FWASIoU) loss is developed to enhance regression of bounding box coordinates for non-rigid wildfire targets.
(4) The proposed ASCA-YOLO model attains an encouraging equilibrium between detection quality and computing cost, which makes it suitable for instantaneous UAV-based wildfire monitoring.

## 2. Methods

### 2.1. YOLO26 Model

Considering both detection performance and deployment efficiency, YOLO26 [39] is adopted as the starting baseline in this study. This choice is motivated by the requirements of UAV-based wildfire monitoring. In practical UAV scenarios, the detector should be lightweight, easy to deploy on edge devices, and capable of low-latency inference. YOLO26 is a recently developed single-stage detector within the YOLO family. Its NMS-free end-to-end architecture reduces post-processing overhead and improves inference consistency, while its lightweight design and training strategy are suitable for real-time edge-oriented applications. These characteristics make YOLO26 a reasonable starting point for exploring wildfire-specific improvements. At the same time, to avoid making the conclusions dependent on a less familiar baseline, this study further compares the proposed ASCA-YOLO with several widely used detectors, as reported in Section 4.3.

**Figure 1:**
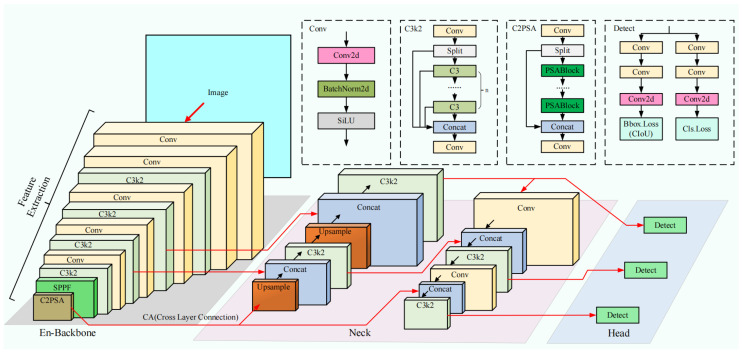
Network Architecture of YOLO26. Solid arrows indicate the direction of the data and feature flow.

The YOLO26 architecture contains three main constituents, namely the Backbone, Neck, and Head. The Backbone is used to extract hierarchical feature representations through stacked Conv and C3k2 modules. The Neck improves feature aggregation by integrating the SPPF and C2PSA modules. The Head conducts multi-scale detection through three parallel branches. The overall network architecture is presented in Figure 1.

### 2.2. ASCA-YOLO Model

#### 2.2.1. FWAMSConv Module

Under high-altitude UAV perspectives, fire spots and smoke usually appear as small-scale targets in the image. In the resized 640 × 640 input images used in this study, many annotated fire and smoke instances have bounding boxes smaller than 32 × 32 pixels, while smoke often appears as thin elongated structures with weak visual contrast. As a result, directly applying YOLO26 on edge devices may lead to an equilibrium between lightweight design and small-target detection capability.

**Figure 2:**
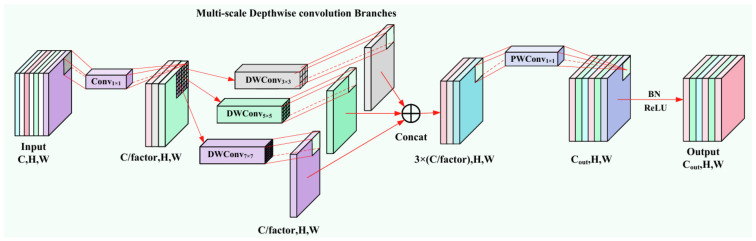
Structure of FWAMSConv. Solid arrows indicate the direction of the feature flow, while dashed lines represent the corresponding channel separation or mapping operations.

To alleviate the issue, a Forest Wildfire Adaptive Multi-Scale Convolution (FWAMSConv) module is proposed to replace the standard convolution in YOLO26. It should be noted that FWAMSConv follows the established design philosophy of multi-branch receptive-field modeling and depthwise separable convolution. The purpose of this module is to provide a lightweight adaptation for wildfire images. The structure of FWAMSConv is presented in Figure 2.

FWAMSConv takes the input feature map as follows:

(1) $$X\in R^{C\times H\times W}$$

To reduce model complexity and improve feature compactness before multi-scale extraction, the block uses a 1 × 1 convolution to reduce the channel dimension, yielding a compressed feature representation:

(2) $$X_{s}={Conv}_{1\times 1}(X)$$

(3) $$X_{s}\in R^{\frac{C}{factor}\times H\times W}$$

This step reduces computational cost by decreasing the channel dimension while preserving the primary semantic information, thereby providing a more compact feature representation for the subsequent multi-scale feature extraction.

Thereafter, the compressed characteristic $X_{s}$ is simultaneously feed into three depthwise separable convolution branches with different receptive fields for multi-scale feature extraction. Compared with standard convolutions, *DW*Conv can accomplish spatial feature extraction with an extremely low number of parameters and floating point operations:

(4) $$X_{3\times 3}=DW{Conv}_{3\times 3}\left(X_{s}\right)$$

(5) $$X_{5\times 5}=DW{Conv}_{5\times 5}\left(X_{s}\right)$$

(6) $$X_{7\times 7}=DW{Conv}_{7\times 7}\left(X_{s}\right)$$

(7) $$X_{3\times 3},X_{5\times 5},X_{7\times 7}\in R^{\frac{C}{factor}\times H\times W}$$

Convolution kernels of different scales correspond to distinct receptive fields, where small kernels emphasize local details, while larger kernels capture richer contextual information, allowing the framework to perceive both subtle flame specifics and broader smoke diffusion patterns.

Subsequently, the features extracted by the three scale paths are concatenated across the channel axis:

(8) $$X_{c}=Concat\left(X_{3\times 3},X_{5\times 5},X_{7\times 7}\right)$$

(9) $$X_{c}\in R^{3\times \frac{C}{factor}\times H\times W}$$

This operation integrates information from receptive fields of different scales, enabling the network to simultaneously encompass multi-scale fire semantic features under a shared representation space, thus boosting the model’s representation capability for complex target structures.

Finally, channel fusion is performed on the concatenated features via a PWConv:

(10) $$X_{o}=PW{Conv}_{1\times 1}\left(X_{c}\right)$$

The final output features are then obtained through BatchNorm and the *ReLU* activation function:

(11) $$\mathrm{Output}=ReLU\left(BN\left(X_{o}\right)\right)$$

(12) $$\mathrm{Output}\in R^{C_{\mathrm{out}}\times H\times W}$$

This fusion process achieves channel reconstruction while preserving multi-scale information, enabling flame and smoke features of different scales to be effectively integrated within a unified feature space, thereby boosting the comprehensive feature representation capability of the model.

Through the aforementioned mechanisms, FWAMSConv, while substantially reducing computational consumption, leverages parallel multi-scale convolutional branches (3 × 3, 5 × 5, and 7 × 7) to optimize the framework’s feature representation performance for extremely small fire spots and smoke, achieving the synchronous perception of local details and macroscopic contextual information.

#### 2.2.2. FWSCSAttention Mechanism

Forest scenes are often affected by background interference, since reflections from sunlight, highly illuminated objects, and light mist can all look similar to fire in images. These factors can introduce false responses during feature extraction and lead to pseudo-fire detections.

**Figure 3:**
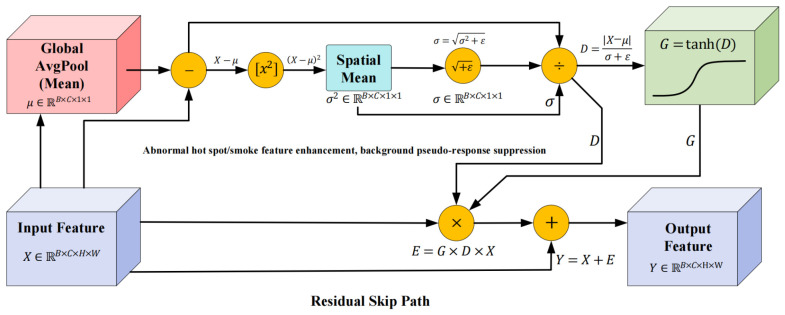
Structure of FWSCSAttention. Solid arrows indicate the direction of the data and feature flow.

To alleviate this problem, a Forest Wildfire Sparse Contextual Saliency Attention (FWSCSAttention) mechanism is introduced. FWSCSAttention is positioned as a task-specific contextual saliency modeling strategy rather than a new general-purpose attention paradigm. Its design is motivated by the observation that fire-related regions often appear as local statistical deviations from the surrounding forest background. By using standardized feature deviations to construct saliency weights, the module aims to enhance fire/smoke-related responses while avoiding additional learnable parameters. The structure is presented in Figure 3.

The input feature map is defined as:

(13) $$X\in R^{B\times C\times H\times W}$$

where B, C, H, and W represent the batch size, the channel count, and the spatial dimensions of the feature map, respectively.

Global average pooling is applied to the input feature X over the spatial dimensions (H, W) to obtain the contextual mean μ for each channel, which is computed as:

(14) μ=Mean(X)

The resulting mean feature has a size of B×C×1×1, representing the average response of each channel over the entire spatial range.

Then, the variance $\sigma^{2}$ of the feature composition is obtained by calculating the squared deviation between the input feature and the mean, followed by spatial averaging, as given by:

(15) $$\sigma^{2}=Mean\left((X-\mu {)}^{2}\right)$$

After the variance is obtained, the standard deviation is computed by applying a square root operation with a stabilization factor ${\varepsilon (\varepsilon =10}^{-6})$:

(16) $$\sigma =\sqrt{\sigma^{2}+\varepsilon }$$

This operation is used to characterize the overall spatial dispersion of the current features.

Next, a standardized deviation metric is constructed to measure the anomaly level of each feature location relative to its contextual statistical distribution:

(17) $$D=\frac{\left|X-\mu \right|}{\sigma +\varepsilon }$$

where D denotes the standardized discrepancy representation, which matches the input feature X in size and reflects the deviation intensity of each spatial location relative to the global statistical distribution.

To further convert the deviation information into saliency weights, a hyperbolic tangent function is used to generate a saliency gate:

(18) G=tanh(D)

where G denotes the adaptive saliency weight map.

Subsequently, the input features are weighted and enhanced through a saliency enhancement operation to obtain the enhanced features:

(19) E=G×D×X

This step jointly modulates the original features through the deviation intensity and the saliency gate, allowing the abnormal response regions to be further amplified.

Finally, to maintain network training stability and avoid destroying the original feature structure, a residual connection is introduced to accomplish feature fusion:

(20) Y=X+E

where Y denotes the resulting feature representation, whose scale remains B×C×H×W.

Through this process, FWSCSAttention suppresses background pseudo-responses by combining contextual statistical modeling with the deviation metric, without introducing additional learnable parameters, thereby improving the anti-interference capability of the model in complex environments.

#### 2.2.3. FWASIoU Loss

Although FWSCSAttention suppresses background interference, existing models still have limitations in bounding box regression for non-rigid targets. Wildfire and smoke usually present dynamic and irregular shapes, which are difficult to model effectively with conventional IoU-based loss functions.

To overcome this limitation, a Forest Wildfire Adaptive Sparse-Aware IoU (FWASIoU) loss is introduced. FWASIoU is an IoU-type regression variant adapted for wildfire detection, rather than a completely new bounding box regression theory. Its design follows the general direction of geometry-aware box regression, but combines center stability, background suppression, and morphological adaptation constraints to better fit the irregular and non-rigid shapes of flame and smoke targets.

The predicted bounding box is represented as $B_{p}=\left(x_{p},y_{p},w_{p},h_{p}\right)$, and the ground truth bounding box is represented as $B_{g}=\left(x_{g},y_{g},w_{g},h_{g}\right)$, where x and y denote the center coordinates of the bounding box, and w and h denote its width and height, respectively. First, the Intersection over Union (IoU) between the predicted box and the ground truth box is computed based on their geometric relationship:

(21) $$IoU=\frac{\left|B_{p}\cap B_{g}\right|}{\left|B_{p}\cup B_{g}\right|}$$

where $\left|B_{p}\cap B_{g}\right|$ represents the intersection area between the predicted box and the ground truth box, $\left|B_{p}\cup B_{g}\right|$ represents their union area.

On this basis, FWASIoU further introduces a center-distance constraint term to improve localization stability for small-scale flame targets. The Euclidean metric is used to measure the distance between the centers of the predicted box and the annotated box:

(22) $$d_{c}^{2}={\left(x_{p}-x_{g}\right)}^{2}+{\left(y_{p}-y_{g}\right)}^{2}$$

Meanwhile, the minimum enclosing rectangle covering both bounding boxes is defined, with width and height denoted by $c_{w}$ and $c_{h}$, respectively. The squared diagonal length of this enclosing box is given by:

(23) $$c_{d}^{2}=c_{w}^{2}+c_{h}^{2}$$

After normalizing the center distance, the center stability constraint term is expressed as:

(24) $$L_{c}=\frac{d_{c}^{2}}{c_{d}^{2}}$$

This term can still provide stable gradients when the IoU value is small, thereby improving localization for extremely small fire-spot targets.

In addition, FWASIoU introduces a bounding consistency constraint to better stabilize feature representation. Let $A_{c}$ denote the area of the minimum enclosing rectangle, and let $A_{u}$ denote the union area of the predicted box and the ground truth box. The background suppression term is described as follows:

(25) $$L_{e}=\frac{A_{c}-A_{u}}{A_{c}}$$

When the predicted box is much larger than the ground truth box or includes a large background region, this term increases noticeably, thereby penalizing the predicted box and encouraging a tighter fit to the true flame region.

To better characterize the non-rigid morphological variations in flames and smoke, FWASIoU further introduces a morphological adaptive constraint term. The morphological difference term is obtained by comparing the aspect-ratio angle difference between the predicted box and the ground truth box:

(26) $$L_{s}={\left(arctan\left(\frac{w_{p}}{h_{p}}\right)-arctan\left(\frac{w_{g}}{h_{g}}\right)\right)}^{2}$$

This term effectively measures the morphological and structural differences between the bounding boxes, enabling the model to better adapt to slender smoke structures and irregular flame contours.

After combining the three constraints described above, the overall expression of FWASIoU can be written as:

(27) $$FWASIoU=IoU-\left(\lambda_{c}L_{c}+\lambda_{s}L_{s}+\lambda_{e}L_{e}\right)$$

where $\lambda_{c},\;{\;\lambda }_{s},\;{\;\lambda }_{e}$ denote the center stability weight, the morphological alignment weight, and the background suppression weight, respectively. In this study, the three weight coefficients are set as $\lambda_{c}=1.0,\;\lambda_{s}=0.5,\;\lambda_{e}=0.5$, and they are kept unchanged in all experiments involving FWASIoU. The center stability term is assigned a relatively higher weight because small fire spots observed from UAV views are sensitive to center-position deviations. The morphological adaptation and background suppression terms are assigned moderate weights to improve the localization of slender smoke and irregular flame regions while avoiding over-penalization of non-rigid or diffused targets.

In this way, FWASIoU, while retaining the foundational overlap optimization, enhances the model’s cross-scene adaptive capability to non-rigid and dynamic fire morphologies by introducing multi-dimensional dynamic geometric constraints. This overcomes the issues of feature representation deviation and localization divergence triggered by target morphological distortions.

#### 2.2.4. ASCA-YOLO

**Figure 4:**
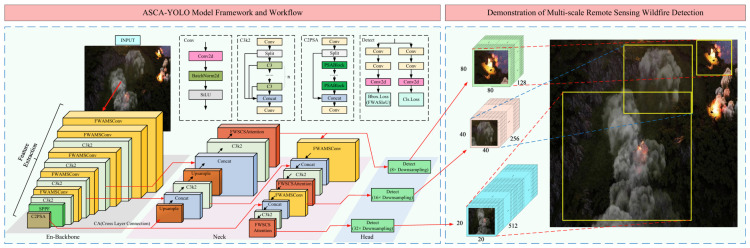
Network Architecture of ASCA-YOLO. Solid arrows indicate the direction of the feature flow, while dashed lines illustrate the corresponding multi-scale feature transformations and detection layer mappings.

By incorporating FWAMSConv, FWSCSAttention, and FWASIoU into the YOLO26 framework, the ASCA-YOLO model is constructed. The proposed model improves feature representation for small fire and smoke targets, which in this study refer to annotated instances with bounding boxes smaller than 32 × 32 pixels. It also suppresses background interference and strengthens bounding box regression for non-rigid fire and smoke structures. The comprehensive network architecture is presented in Figure 4.

In ASCA-YOLO, FWAMSConv replaces the standard convolutional layers in the Backbone and Neck of YOLO26. FWSCSAttention is added after the multi-scale feature fusion layers in the Neck. FWASIoU is used as the bounding box regression loss in the detection head during training. The remaining network structure, including the detection head and input-output settings, follows the original YOLO26 baseline.

## 3. Materials

### 3.1. Dataset

To improve the performance and generalization ability of network training, this paper uses the forest-fire_dataset provided by Roboflow together with the large-scale public UAV remote-sensing forest fire dataset M4SFWD [40]. M4SFWD mainly provides UAV/remote-sensing wildfire images with variations in forest terrain, illumination, weather, and fire-object distribution, while the Roboflow dataset provides additional heterogeneous fire and smoke images from public or real-world visual sources.

**Figure 5:**
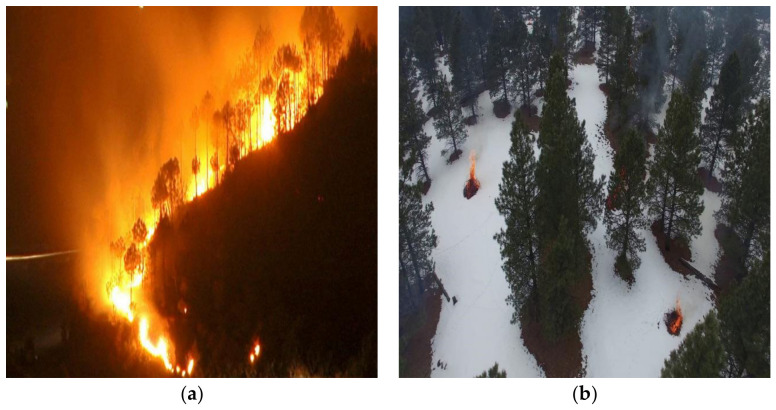
Example images of the forest wildfire dataset. (**a**) Nighttime wildfire in a mountainous forest, northern Portugal (8.25° W, 41.35° N), from the forest-fire_dataset. (**b**) UAV aerial view of sparse fires in a snow-covered coniferous forest, Oregon, USA (122.10° W, 44.05° N), from the M4SFWD dataset.

During dataset integration, all images were first converted to RGB format, and the original annotations were converted into YOLO format. Data cleaning was then performed to remove corrupted images, unreadable images, images with extremely low resolution, severely blurred images, images with unrecognizable targets, and images irrelevant to forest wildfire detection. Exact duplicates were detected using file hashing. Near-duplicate images were screened using perceptual hashing, and candidate image pairs with a Hamming distance no greater than 6 were manually reviewed. For identical or nearly identical images, only the image with better visual quality was retained. After cleaning and filtering, 7414 images were retained in wildfire dataset, including 4312 images from M4SFWD and 3102 images from Roboflow. Representative examples are represented in Figure 5.

Considering that UAV wildfire images may contain similar frames from the same scene, video sequence, or fire event, this study adopted a scene-level/event-level split rather than a simple image-level random split. Specifically, each image was assigned a scene/event group ID according to its data source, original folder, video sequence, scene background, fire event, and near-duplicate screening results. Images from the same video sequence, UAV flight scene, fire event, or near-duplicate group were assigned to only one subset. Finally, the 7414 images were organized into 486 scene/event groups and divided into train, valid, and test sets at an approximate ratio of 7:2:1, containing 5189, 1482, and 743 images, respectively. This splitting strategy was used to reduce the risk of temporal leakage and scene leakage during model evaluation. Scene-level split of the curated wildfire dataset is listed in Table 1.

All retained images were rechecked and re-annotated using LabelImg 1.8.6 into two categories: “fire” and “smoke”. Visible flame regions were labeled as fire, and visible smoke regions were labeled as smoke. Fire and smoke appearing in the same image were annotated separately. During preprocessing, all images were adjusted to a resolution of 640 × 640 pixels. Depending on the specific UAV flight altitude and camera parameters, this resolution corresponds to an approximate ground coverage area ranging from 50 × 50 m^2^ to 200 × 200 m^2^.

### 3.2. Model Running Environment and Parameter Settings

To improve reproducibility, all training settings are specified as follows. All models were trained and evaluated on Windows 11 with an Intel Core i5-14600KF CPU, 32 GB RAM, and an NVIDIA GeForce RTX 5060 Ti GPU with 16 GB VRAM. The software environment included Python 3.10.19, PyTorch 2.10.0, and CUDA 12.8. All images were resized to 640 × 640 pixels. The batch size was 4, and the number of training epochs was 100. Pretrained weights were used for initialization.

MuSGD was adopted as the optimizer, with lr0=0.01, lrf=0.01, momentum=0.937, and weightdecay=0.0005. The warm-up stage lasted for 3 epochs, with warm-up momentum of 0.8 and warm-up bias learning rate of 0.1. Cosine learning rate scheduling was not used. The augmentation strategy included HSV augmentation with hsv_h=0.015, hsv_s=0.7, and hsv_v=0.4, translation of 0.1, scaling of 0.5, horizontal flipping with a probability of 0.5, and Mosaic augmentation with a probability of 1.0. Mosaic augmentation was disabled during the last 10 epochs, while Mixup, CutMix, copy-paste, vertical flipping, rotation, shear, and perspective transformation were not used.

The random seed was set to 0, and deterministic training was enabled. Automatic mixed precision was disabled. During validation, the IoU threshold was 0.7, and the maximum number of detections per image was 300. Since YOLO26 adopts an NMS-free end-to-end detection paradigm, no additional conventional NMS was used. Input images were normalized by scaling pixel values from [0, 255] to [0, 1]. The same data split and training settings were used for all comparative and ablation experiments.

### 3.3. Evaluation Metrics

We assessed the proposed model in terms of both detection performance and computational cost, using six common metrics: precision, recall, mAP50, mAP50-95, parameter count, and floating point operations. The specific descriptions of each metric are as follows.

Precision (P) is used to measure the percentage of true positive categories within the samples predicted as positive categories via the model, reflecting the precision capability of the model. Its calculation formula is:

(28) $$P=\frac{TP}{TP+FP}$$

where TP denotes the number of true targets correctly detected by the model, and FP denotes the number of background regions incorrectly identified as targets.

Recall (*R*) represents the proportion of all true targets that are accurately detected via the model, indicating the model’s capability to reduce missed detections. It is calculated as:

(29) $$R=\frac{TP}{TP+FN}$$

where FN denotes the number of true targets missed by the model.

mAP50 is the mean Average Precision calculated for all target classes under an IoU threshold of 0.5, and it illustrates the overall quality of the model in target recognition and localization. It is expressed as:

(30) $$mAP50=\frac{1}{N}\sum_{i=1}^{N}\int_{0}^{1}P_{i}(R_{i})dR_{i}$$

where N is the total number of target categories in the dataset (in this study, N=2), and $P_{i}(R_{i})$ denotes the smoothed precision-recall curve function for the i=th category.

mAP50-95 is used to measure the arithmetic mean of the mAP under a total of 10 different thresholds as the IoU threshold increases ranging from 0.5 to 0.95 in increments of 0.05, which serves as a rigorous assessment of the model’s high-precision bounding box regression capability. Its calculation formula is:

(31) $$mAP50-95=\frac{1}{10}\sum_{IoU=0.5:0.95}^{0.95}{mAP}_{IoU}$$

where ${mAP}_{IoU}$ denotes the mean Average Precision under the specific IoU threshold requirement.

Parameter count (Par) is used to measure the overall number of learnable parameters contained in the model’s network architecture, straightforwardly reflecting the storage requirement of the model on edge devices and its spatial complexity.

Floating point operations (FLOPs) is used to measure amount of billion floating point operations required by the model to process a single input image, intuitively reflecting the computational complexity and inference speed potential of the model.

Frames per second (FPS) is used to measure the number of images that the model can process per second during inference, directly reflecting the real-time detection capability of the model under a given hardware environment. A higher FPS indicates faster inference speed and better potential for real-time UAV-based wildfire monitoring.

Missed detection rate (MDR) is used to measure the proportion of true targets that are not detected by the model, reflecting the missed detection risk of the model in wildfire monitoring. Its calculation formula is:

(32) $$MDR=\frac{FN}{TP+FN}=1-R$$

False alarm rate (FAR) is used to measure the proportion of false positive detections among all predicted positive detections, reflecting the false alarm risk of the model under complex background conditions. Its calculation formula is:

(33) $$FAR=\frac{FP}{TP+FP}=1-P$$

## 4. Results and Discussion

### 4.1. Comparative Experiments of Different Modules

#### 4.1.1. Comparative Experiment of Convolutional Modules

To evaluate the effectiveness of Forest Wildfire Adaptive Multi-Scale Convolution (FWAMSConv) in extracting small-scale features under limited computational cost, comparative experiments are conducted against the baseline YOLO26 and several representative convolutional modules, including DWConv, GhostConv, PConv, and FSConv. The quantitative results are presented in Table 2.

Due to structural limitations, conventional convolutional modules often face challenges in balancing computational efficiency and fine-grained feature representation. DWConv reduces computational cost through channel-wise decomposition, lowering FLOPs to 3.6 G. However, its limited receptive field restricts contextual modeling, which may lead to the loss of small-scale fire features during deep feature extraction. As a result, the recall decreases to 0.748. GhostConv reduces parameter redundancy by generating feature maps through linear transformations, decreasing the parameter count to 2.050 M. However, this mechanism may weaken fine-grained edge representations, particularly for small flame regions, which can limit detection performance. Consequently, mAP50 remains at 0.838. PConv reduces memory access by using convolution operations to a selection of input channels. However, this partial channel processing strategy may limit cross-channel feature interaction, resulting in increased computational cost rather than reduction. In this study, FLOPs reach 6.0 G, indicating reduced efficiency. FSConv introduces frequency-spatial feature fusion to improve representation capability. However, the increased computational complexity leads to FLOPs of 6.3 G. In addition, its limited ability to suppress background interference may result in performance degradation, with recall decreasing to 0.735 and mAP50 to 0.819.

In contrast, FWAMSConv improves feature representation through channel compression and parallel multi-scale depthwise separable branches. The model achieves a parameter count of 1.870 M, FLOPs of 4.2 G and FPS of 119.7, indicating improved efficiency. At the same time, P, R, mAP50, and mAP50-95 reach 0.843, 0.784, 0.861, and 0.538, respectively. The improved recall indicates that the model becomes more effective in detecting small fire spots and slender smoke, including small-scale instances with bounding boxes smaller than 32 × 32 pixels. Correspondingly, the MDR decreases from 0.235 for YOLO26 to 0.216 for YOLO26 + FWAMSConv, further indicating that FWAMSConv helps reduce missed detections of weak wildfire targets. Taken together, these results demonstrate that FWAMSConv attains a better equilibrium between computational expense and detection effectiveness in complex wildfire scenarios.

**Figure 6:**
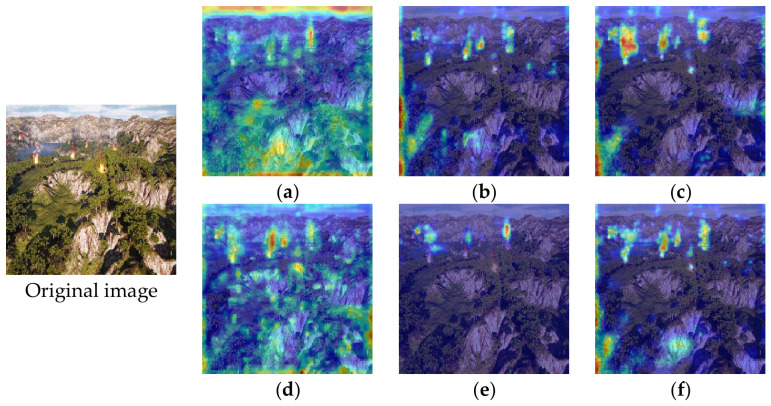
Heatmaps of different convolutional modules, areas of elevated brightness correspond to regions prioritized by the network. Aerial view of multi-spot wildfires in a rocky mountainous forest, Cantabria, northern Spain (4.12° W, 43.23° N), from the M4SFWD dataset. (**a**–**f**) represent the YOLO26, YOLO26 + DWConv, YOLO26 + GhostConv, YOLO26 + PConv, YOLO26 + FSConv, and YOLO26 + FWAMSConv models, respectively.

To further examine the feature extraction behavior of different convolutional modules, Grad-CAM is employed to visualize deep feature responses. An image containing multiple small fire spots and slender smoke structures is selected to compare the attention distributions of diverse models, and the corresponding results are presented in Figure 6.

The visualization results indicate that YOLO26 and the models using conventional convolutional modules exhibit relatively scattered activation patterns. Some small fire regions, especially those located near the image boundaries, are not highlighted consistently. By contrast, the model integrated with FWAMSConv produces more concentrated activation responses around small flame regions and thin smoke structures. This finding implies that FWAMSConv enhances feature localization for small-scale targets. Overall, the visualization results are in agreement with the quantitative results, showing that the proposed module improves feature representation for small and sparse wildfire targets while preserving computational efficiency.

#### 4.1.2. Comparative Experiment of Attention Mechanisms

To evaluate the effectiveness of FWSCSAttention in complex environments, comparative experiments are conducted with the baseline YOLO26 and several representative attention mechanisms, including CBAM, LCA, DynamicSpatialAttention, and HPAttention. The results are presented in Table 3.

As shown in Table 3, conventional attention mechanisms do not consistently improve detection performance in complex forest environments. CBAM relies on global max pooling to infer spatial attention. However, strong local responses caused by background interference may be amplified by the max pooling operation, leading to incorrect activations. This results in decreased performance, with P and R dropping to 0.818 and 0.759, respectively. LCA and DynamicSpatialAttention focus on modeling local spatial relationships or dynamic convolutional features. However, these methods may exhibit limited generalization when dealing with diverse wildfire scenarios. In particular, DynamicSpatialAttention shows a decrease in recall to 0.742, indicating reduced sensitivity to true fire targets. HPAttention introduces hierarchical pooling for feature fusion. However, it does not sufficiently separate fire-related features from background interference, which may introduce redundant feature responses. As a result, mAP50 decreases to 0.839.

**Figure 7:**
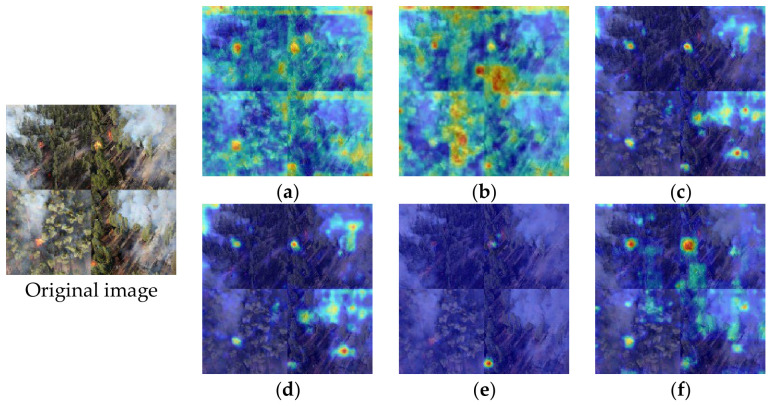
Heatmaps of different attention mechanisms; areas of elevated brightness correspond to regions prioritized by the network. Aerial view of dense coniferous forest fires with heavy smoke, British Columbia, western Canada (120.5° W, 51.2° N), from the forest-fire_dataset. (**a**–**f**) represent the YOLO26, YOLO26 + CBAM, YOLO26 + LCA, YOLO26 + DynamicSpatialAttention, YOLO26 + HPAttention, and YOLO26 + FWSCSAttention models, respectively.

In contrast, FWSCSAttention enhances feature discrimination through contextual statistical modeling. The model reaches 0.841 in precision, 0.786 in recall, 0.861 in mAP50, and 0.540 in mAP50-95. The gain in precision indicates fewer false positives when the background is complex. Correspondingly, the FAR decreases from 0.173 for YOLO26 to 0.159 for YOLO26 + FWSCSAttention, further confirming that FWSCSAttention can reduce false alarms caused by fire-like background interference. This suggests that the attention mechanism helps the model remain more stable in challenging scenes. To further examine the behavior of different attention mechanisms, the corresponding visualization results are presented in Figure 7.

The visualization results indicate that YOLO26 and the models using conventional attention mechanisms generate noticeable activations in non-fire regions, revealing their sensitivity to background interference. In contrast, the model equipped with FWSCSAttention exhibits more concentrated responses in actual fire and smoke regions while suppressing irrelevant background activations. This suggests stronger feature discrimination in complex scenes.

#### 4.1.3. Comparative Experiment of Loss Functions

To evaluate the performance of FWASIoU in dealing with non-rigid targets, comparative experiments are performed with the baseline YOLO26 and several representative loss functions, including SDIoU, SIoU, WIoU, and MPDIoU. The corresponding results are reported in Table 4.

Since the loss function mainly affects the training process, the parameter count and FLOPs remain unchanged across all models. Conventional IoU-based loss functions often struggle to handle the irregular and dynamic shapes of wildfire targets. As a result, performance degradation is observed in several cases. SDIoU and MPDIoU improve bounding box alignment by introducing distance-based constraints. However, wildfire targets often lack stable geometric reference points due to their irregular shapes. As a result, these methods show limited adaptability, leading to mAP50 values of 0.838 and 0.834, respectively. SIoU introduces an angle-based constraint to improve convergence. However, this rigid geometric constraint may not be well suited to highly dynamic and amorphous wildfire targets. Consequently, recall decreases to 0.748, indicating reduced localization stability. WIoU adopts a dynamic focusing mechanism to balance gradients between easy and hard samples. However, without explicit geometric adaptation for target deformation, the regression process may become unstable, resulting in a decrease in precision to 0.822.

In contrast, FWASIoU introduces multiple constraints, including center stability, bounding consistency, and morphological adaptation. The model achieves P, R, mAP50, and mAP50-95 of 0.854, 0.779, 0.866, and 0.542, respectively. The improvement in mAP50-95 suggests more stable localization for irregular wildfire targets.

**Figure 8:**
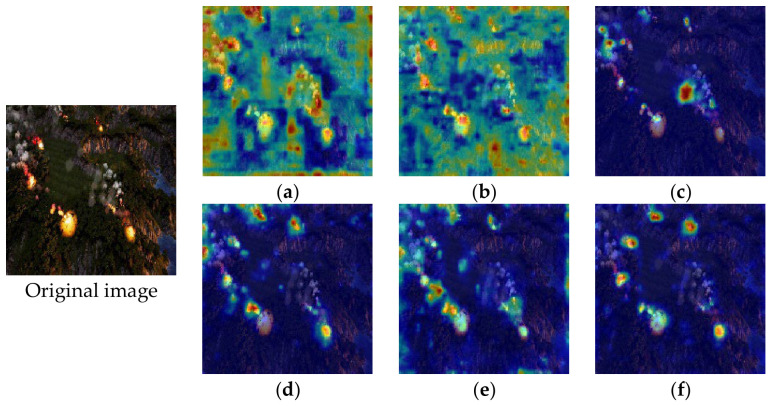
Heatmaps of different loss functions, areas of elevated brightness correspond to regions prioritized by the network. Aerial view of clustered wildfires in a mountainous coastal forest, Attica, Greece (23.8° E, 38.1° N), from the M4SFWD dataset. (**a**–**f**) represent the YOLO26, YOLO26 + SDIoU, YOLO26 + SIoU, YOLO26 + WIoU, YOLO26 + MPDIoU, and YOLO26 + FWASIoU models, respectively.

To further analyze the effect of different loss functions, Grad-CAM is used to visualize feature responses for non-rigid wildfire targets. An image containing diffused smoke and irregular fire structures is selected, and the results are presented in Figure 8.

The visualization results indicate that YOLO26 and the models using conventional loss functions exhibit dispersed activation patterns, with limited alignment to irregular target boundaries. In contrast, the model using Forest Wildfire Adaptive Sparse-Aware IoU shows more concentrated responses along the contours of fire and smoke. The result points to better localization performance for non-rigid targets.

### 4.2. Ablation Experiment

We conducted ablation experiments on the wildfire dataset to assess the role of each module. The results are listed in Table 5.

Using YOLO26 alone (Model 1), the model reaches 0.827 precision, 0.765 recall, 0.846 mAP50, and 0.512 mAP50-95, leaving clear room for further improvement in both accuracy and stability. After adding FWAMSConv (Model 2), recall and mAP50 rise to 0.784 and 0.861, while both FLOPs and parameter count drop, showing that this module improves efficiency and benefits small-target detection. With only FWSCSAttention (Model 3), mAP50-95 increases to 0.540, which reflects better resistance to background interference. Using FWASIoU alone (Model 4) also leads to gains in precision and mAP, pointing to more accurate localization for non-rigid targets.

The paired combinations also show clear interaction effects. FWAMSConv together with FWSCSAttention (Model 5) improves both precision and recall. FWAMSConv combined with FWASIoU (Model 6) strengthens small-target detection and localization, although its resistance to background clutter is still limited. FWSCSAttention plus FWASIoU (Model 7) further improves robustness and localization, but the computational burden remains comparatively high.

The best overall result appears in Model 8, where all three modules are used together. In this setting, ASCA-YOLO achieves 0.870 precision, 0.809 recall, 0.895 mAP50, 0.578 mAP50-95 and 122.5 FPS. Compared with YOLO26, all evaluation metrics improve, while FLOPs and parameter count are both reduced. In addition, ASCA-YOLO reduces the MDR to 0.191 and the FAR to 0.130, indicating fewer missed detections and false alarms under the joint effect of the three modules. This indicates that the three modules work well together and enhance detection performance without increasing computational cost.

### 4.3. Comparative Experiment with Other Detection Models

We further compared the proposed model with several widely used object detection methods, and the results are shown in Table 6.

Faster R-CNN, as a typical two-stage detector, relies on region proposals before final prediction. This design helps improve localization, but it also brings a heavy computational burden. In our experiments, Faster R-CNN reaches the highest recall at 0.859, yet its 181.4 G FLOPs and 41.3 M parameters make it less practical for UAV platforms with limited onboard resources. SSD detects objects from multi-scale feature maps, but its representation of small targets is still limited. This is reflected in its recall of 0.565, suggesting weaker sensitivity to small wildfire regions. RetinaNet achieves a high precision of 0.876 by using Focal Loss to alleviate class imbalance, but its computational cost remains high, with FLOPs reaching 256.9 G. RT-DETR benefits from global feature interaction through self-attention. However, its computational complexity increases with image resolution, which may result in high resource consumption when processing UAV-based remote sensing data.

Single-stage YOLO-based models provide advantages in computational efficiency due to their end-to-end architectures. Even so, conventional YOLO variants still depend on standard convolution and IoU-based loss design, which can limit their performance when small targets and complex backgrounds appear at the same time. This is also reflected in their relatively constrained precision and mAP50-95.

By comparison, ASCA-YOLO improves both accuracy and efficiency. It achieves 0.895 mAP50 and 0.578 mAP50-95, while reducing FLOPs and parameter count to 4.2 G and 1.870 M. In addition, ASCA-YOLO reaches an inference speed of 122.5 FPS, indicating that the model can maintain high detection accuracy while providing sufficient real-time processing capability. Among the compared models, it offers a more balanced trade-off between detection quality and computational cost. Although it is not the best in every single metric, its overall behavior is more stable and better suited to practical UAV wildfire monitoring.

**Figure 9:**
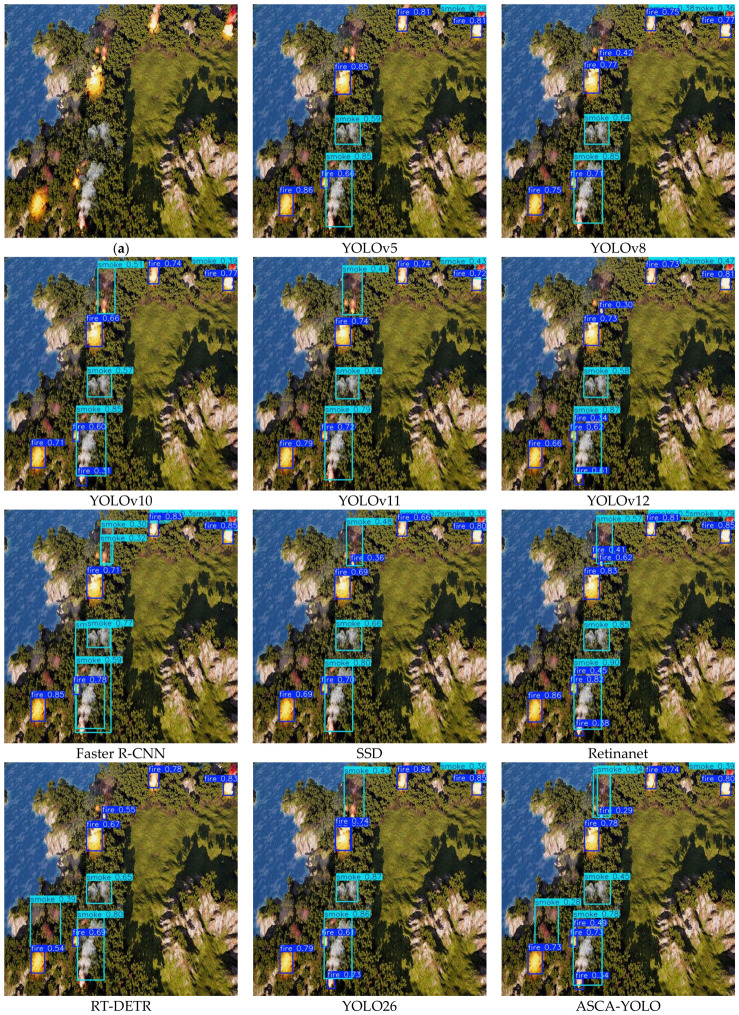
Performance assessment of distinct object detectors presented in Figure (**a**).

**Figure 10:**
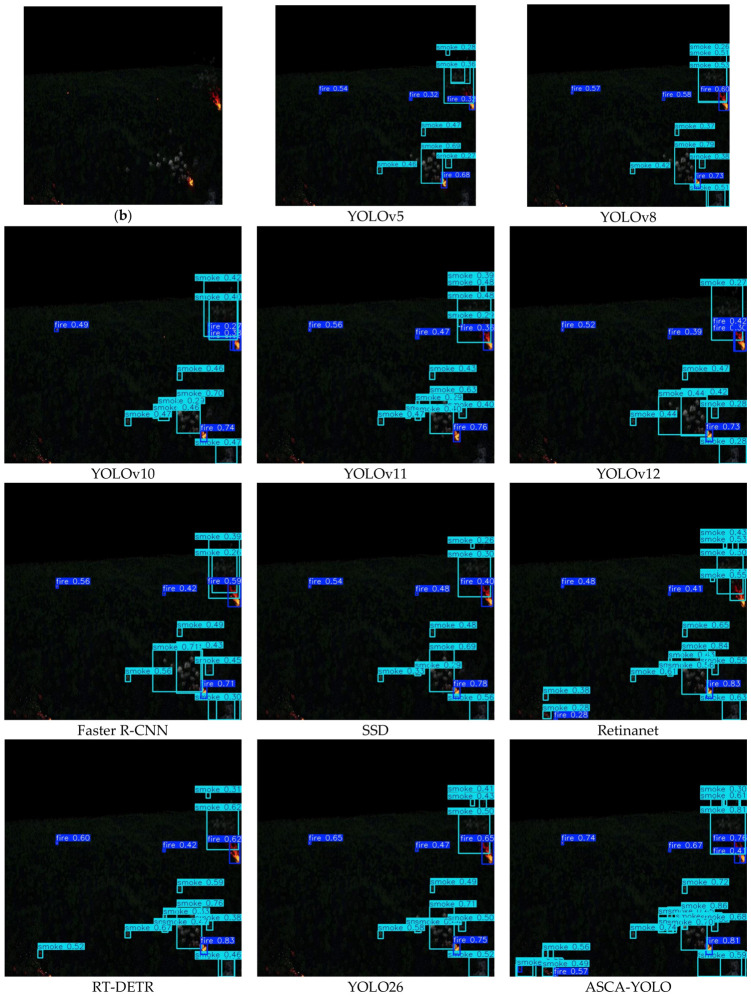
Performance assessment of distinct object detectors presented in Figure (**b**).

To further examine model behavior in difficult cases, we also provide qualitative results on representative test images. Two typical scenarios are considered: small fire targets and dispersed smoke under dark background conditions. The results are shown in Figure 9 and Figure 10.

In Figure (a), which contains small and sparse fire targets, several models exhibit missed detections to varying degrees. In contrast, ASCA-YOLO is able to detect most of the visible fire and smoke regions. This suggests improved sensitivity to small-scale targets and a reduced missed detection rate for all targets. In Figure (b), which contains complex background interference, several models produce either missed detections or incorrect activations. In comparison, ASCA-YOLO shows more consistent detection results, with reduced false responses in non-fire regions and improved identification of smoke targets. Overall, the qualitative and quantitative results are consistent. The proposed model improves the recognition of minor wildfire objects and reduces the impact of background distraction, while maintaining computational efficiency. These results indicate that ASCA-YOLO is suitable for UAV-based wildfire detection in complex environments.

### 4.4. Deployment Test on a Raspberry Pi Platform

To further assess the practical deployment potential of ASCA-YOLO in resource-constrained UAV scenarios, a brief deployment test was conducted on a Raspberry Pi platform. Since inference performance on embedded hardware may differ considerably from that on a desktop GPU, this experiment was intended to provide a preliminary device-level evaluation of runtime efficiency and resource consumption.

In this experiment, the trained YOLO26 and ASCA-YOLO models were deployed on a Raspberry Pi 4 platform equipped with 8 GB RAM and running Raspberry Pi OS (64-bit). PyTorch was used as the inference framework, and the input resolution was fixed at 640 × 640, consistent with the settings used in the main experiments. During evaluation, single-image inference with a batch size of 1 was adopted. The reported indicators included average inference latency per image, frames per second (FPS), and peak memory usage. Each result was obtained by averaging over 200 test images.

The deployment results are presented in Table 7. On the Raspberry Pi 4 platform, YOLO26 achieved an average inference latency of 952.4 ms per image and a throughput of 1.05 FPS, with a peak memory usage of 735 MB. In comparison, ASCA-YOLO achieved an average inference latency of 826.7 ms per image and a throughput of 1.21 FPS, while reducing peak memory usage to 668 MB. These results indicate that the proposed model imposes a lower deployment burden than the baseline model under embedded conditions. Although the inference speed on the Raspberry Pi 4 remained significantly lower than that observed on the desktop GPU, ASCA-YOLO was still able to perform stable forward inference within limited hardware resources.

The preliminary deployment results on Raspberry Pi 4 show that ASCA-YOLO can run stably on an embedded platform with limited hardware resources. These results further support its deployment feasibility for UAV-based wildfire monitoring and demonstrate its potential engineering application value on edge-oriented devices.

## 5. Conclusions

This study addresses two key challenges in UAV-based forest wildfire detection: the difficulty of capturing small-scale features under limited computational resources and the limited robustness to background interference and non-rigid target structures. To this end, an improved single-stage detection model, ASCA-YOLO, is proposed. After adding FWAMSConv, FWSCSAttention, and FWASIoU, the model enhances feature representation, suppresses background interference, and improves bounding box regression.

The results show that ASCA-YOLO provides a better trade-off between detection performance and computational cost. Compared with the baseline and other representative detectors, it improves the main evaluation results while keeping both parameter count and FLOPs lower. The FPS improvement further reduces inference latency, which is beneficial for timely fire and smoke detection in UAV-based monitoring. The gains in recall and mAP50-95 also suggest better sensitivity to small wildfire targets and better adaptation to irregular fire and smoke patterns.

The proposed method performs well in the current experiments and has also shown preliminary deployment feasibility on the Raspberry Pi 4 platform. However, the present deployment validation remains limited to a brief embedded test. Future work will focus on further deployment and optimization on the NVIDIA Jetson Nano platform, with particular attention paid to real-time performance under practical constraints such as limited power supply, varying environmental conditions, and dynamic flight scenarios. These efforts are expected to support the development of reliable and efficient UAV-based wildfire monitoring systems.

## Figures and Tables

**Table 1:** Scene-level split of the curated wildfire dataset.

Subset	Scene/Event Groups	Images	Ratio
Train	384	5189	69.99%
Valid	67	1482	19.99%
Test	35	743	10.02%
Total	486	7414	100%

**Table 2:** Performance Results of Different Convolutional Modules.

Model	P	R	mAP50	mAP50-95	FLOPs (G)	Par (M)	FPS
YOLO26	0.827	0.765	0.846	0.512	5.2	2.375	106.4
YOLO26 + DWConv [41]	0.821	0.748	0.835	0.503	**3.6**	**1.713**	**135.8**
YOLO26 + GhostConv [42]	0.829	0.760	0.838	0.508	4.4	2.050	115.1
YOLO26 + PConv [43]	0.832	0.758	0.843	0.511	6.0	2.251	84.9
YOLO26 + FSConv [44]	0.814	0.735	0.819	0.482	6.3	2.238	61.5
YOLO26 + FWAMSConv	**0.843**	**0.784**	**0.861**	**0.538**	4.2	1.870	119.7

**Table 3:** Performance Results of Different Attention Mechanisms.

Model	P	R	mAP50	mAP50-95	FLOPs (G)	Par (M)	FPS
YOLO26	0.827	0.765	0.846	0.512	5.2	2.375	106.4
YOLO26 + CBAM [45]	0.818	0.759	0.839	0.508	5.3	2.462	107.4
YOLO26 + LCA [46]	0.819	0.761	0.836	0.503	5.2	2.464	112.9
YOLO26 + DynamicSpatialAttention [47]	0.831	0.742	0.832	0.500	5.3	2.466	110.1
YOLO26 + HPAttention [48]	0.821	0.760	0.839	0.502	5.3	2.376	93.5
YOLO26 + FWSCSAttention	**0.8** **41**	**0.** **786**	**0.8** **61**	**0.5** **40**	**5.2**	**2.375**	**114.0**

**Table 4:** Performance Results of Different Loss Functions.

Model	P	R	mAP50	mAP50-95	FLOPs (G)	Par (M)	FPS
YOLO26	0.827	0.765	0.846	0.512	5.2	2.375	106.4
YOLO26 + SDIoU [49]	0.820	0.762	0.838	0.503	5.2	2.375	106.9
YOLO26 + SIoU [50]	0.832	0.748	0.836	0.510	5.2	2.375	107.1
YOLO26 + WIoU [51]	0.822	0.771	0.848	0.512	5.2	2.375	106.1
YOLO26 + MPDIoU [52]	0.821	0.756	0.834	0.505	5.2	2.375	**107.4**
YOLO26 + FWASIoU	**0.8** **54**	**0.** **779**	**0.8** **66**	**0.5** **42**	**5.2**	**2.375**	106.5

**Table 5:** Results of the Improved Ablation Experiment. The “√” symbol indicates that the corresponding module is included in the model configuration.

Model	Baseline	FWAMSConv	FWSCSAttention	FWASIoU	P	R	mAP50	mAP50-95	FLOPs (G)	Par (M)	FPS
1	YOLO26				0.827	0.765	0.846	0.512	5.2	2.375	106.4
2		√			0.843	0.784	0.861	0.538	4.2	1.870	119.7
3			√		0.841	0.786	0.861	0.540	5.2	2.375	114.0
4				√	0.854	0.779	0.866	0.542	5.2	2.375	106.5
5		√	√		0.860	0.792	0.872	0.552	4.2	1.870	122.1
6		√		√	0.853	0.781	0.867	0.541	4.2	1.870	119.6
7			√	√	0.856	0.788	0.871	0.551	5.2	2.375	118.4
8		√	√	√	**0.8** **70**	**0.8** **09**	**0.** **895**	**0.** **578**	**4.2**	**1.870**	**122.5**

**Table 6:** Results of the Comparative Experiments.

Model	P	R	mAP50	mAP50-95	FLOPs (G)	Par (M)	FPS
YOLOv5	0.838	0.764	0.851	0.510	7.1	2.503	80.7
YOLOv8	0.839	0.775	0.854	0.520	8.1	3.006	96.5
YOLOv10	0.818	0.748	0.826	0.506	6.5	2.266	98.7
YOLOv11 [53]	0.832	0.781	0.858	0.526	6.3	2.583	105.0
YOLOv12	0.829	0.770	0.848	0.513	6.3	2.557	111.4
Faster R-CNN	0.809	**0.859**	0.860	0.524	181.4	41.3	55.1
SSD	0.836	0.565	0.718	0.451	30.5	13.872	74.0
Retinanet [54]	**0.876**	0.798	0.875	0.562	256.9	36.37	48.3
RT-DETR [55]	0.852	0.805	0.864	0.535	103.4	31.988	56.5
YOLO26	0.827	0.765	0.846	0.512	5.2	2.375	106.4
ASCA-YOLO	0.870	0.809	**0.** **895**	**0.** **578**	**4.2**	**1.870**	**122.5**

**Table 7:** Test results of YOLO26 and ASCA-YOLO.

Model	Latency (ms)	FPS	Peak Memory (MB)
YOLO26	952.4	1.05	735
ASCA-YOLO	826.7	1.21	668

## Data Availability

The data presented in this study are available upon request from the corresponding author due to the ongoing nature of this research.

## Abbreviations

The following abbreviations are used in this manuscript:

ASCA-YOLO	Adaptive Sparse and Context-Aware YOLO
FWAMSConv	Forest Wildfire Adaptive Multi-Scale Convolution
FWSCSAttention	Forest Wildfire Sparse Contextual Saliency Attention
FWASIoU	Forest Wildfire Adaptive Sparse-Aware IoU
UAV	Unmanned Aerial Vehicle
SPPF	Spatial Pyramid Pooling Fast
ReLU	Rectified Linear Unit
BatchNorm	Batch Normalization
P	Precision
R	Recall
mAP50	mean Average Precision at an IoU threshold of 0.5
mAP50-95	mean Average Precision at IoU thresholds from 0.5 to 0.95
Par	Parameter Count
FLOPs	Floating Point Operations
FPS	Frames Per Second
MDR	Missed detection rate
FAR	False alarm rate
